# When the guns fall silent… Priorities for health in post-war Ukraine

**DOI:** 10.1093/eurpub/ckac090

**Published:** 2022-08-01

**Authors:** Martin McKee, Iveta Nagyova

**Affiliations:** Department of Health Services Research and Policy, London School of Hygiene & Tropical Medicine, London, UK; European Public Health Association—EUPHA, Utrecht, The Netherlands; European Public Health Association—EUPHA, Utrecht, The Netherlands; Department of Social and Behavioural Medicine, Faculty of Medicine, European Public Health Association, PJ Safarik University, Kosice, Slovakia

The Ukrainian people have suffered enormously from illegal aggression by Russia, whose forces have devastated large parts of the country and committed numerous war crimes.^1^ We may never know how many have been killed but it is certainly in the tens of thousands. Many more will be left with life-changing injuries, both physical and mental. Even larger numbers have left Ukraine, moving westwards to seek sanctuary or, worryingly, in forced deportations to Russia. Colossal damage has been caused by Russian targeting of essential infrastructure, including hundreds of health facilities, and by indiscriminate shelling of urban areas. But some day, hopefully soon, the war will be over and the task of reconstruction will begin.

The cost will be enormous. In early May 2022, President Zelensky suggested a figure of $600 billion. By now, we can assume it will be much higher. And it will take years. There will be many demands on the Ukrainian authorities so it will be necessary to prioritize. While the decisions about what to prioritize will, rightly, be a matter for the Ukrainian government, we can identify several lessons from other conflicts in recent decades.

The first is the need for a robust system of governance. Unfortunately, as a recent systematic review revealed, this is an issue that has received far too little attention in recent conflicts.^2^ Yet there are lessons from the research that exists, including the crucial importance of coordination (often complicated by competition among donors and recipients), the recognition of power imbalances and the importance of understanding context. The need for robust governance systems was apparent in post-invasion Iraq. As was the case there, the recovery effort in Ukraine will act as a magnet for unscrupulous companies and individuals. Ukraine has made considerable progress since 2014 in tackling what was pervasive corruption. However, the vast sums of money involved and the imperative to move quickly will place the safeguards that have been created under pressure, just as was seen with the scandals when some western countries purchased often useless supplies at vastly inflated prices during the coronavirus disease 2019 pandemic.^3^ The principles of public procurement are clear, including transparency and value for money. Urgency is not an excuse to set them aside.

The second is the need to look for innovative ways in rebuilding health systems. Even if it was clinically appropriate to rebuild the many health facilities that have been damaged, which it often would not be given the many ways that the delivery of care has changed since they were built, it would be very difficult to do so. Even acquiring the steel and concrete needed will overwhelm available manufacturing capacity and health providers will have to compete with other sectors such as transport. Before it was destroyed, the Azovstal metallurgical factory in Mariupol was one of the Europe’s largest, producing over 4 million tonnes of steel each year. Some modern, technologically sophisticated facilities will be necessary but this is an opportunity to develop new models of community-based care that is appropriate for the needs of Ukrainians with chronic mental and physical conditions.

The third is innovative responses to labour shortages that will also be required. This is a problem facing all of Europe but exacerbated in a country where so many have fled, particularly young women who previously contributed disproportionately to the health workforce. These responses should draw on the growing body of evidence on task shifting, establishing new types of relationships between health professionals, patients and their carers, and, increasingly, technology.^4^ This will, however, require investment in the co-creation of solutions with all involved, recognizing the challenges posed by longstanding beliefs about professional roles and the low status of nurses in Ukraine.

Finally, there will be a number of specific priorities. Many have been set out in a WHO document which, at the time of writing, has been issued for consultation.^5^ It contains a series of ‘do’s and don’ts’ to achieve a person-centred health system. One is an investment in public health and emergency preparedness and response. A Joint External Evaluation of Ukraine’s implementation of the International Health Regulations, conducted in 2021, provides guidance on what is needed to create a modern health security system. Others cover the delivery of health services, with an emphasis on modern primary care, the health workforce, including training in how best to meet the complex needs of conflict-affected populations, and renewing health infrastructure, including embracing the opportunities offered by advances in digital technology. Other topics covered in the document include reform of health financing and governance.

None of this will be easy. One of us was in besieged Sarajevo in the early 1990s, working with Bosnian colleagues to prepare for the post-war reconstruction. Even then, many of the principles outlined above were clear. But the fragmented state that emerged from the Dayton agreement struggled to establish a shared vision or a robust system of governance to implement it. After all that the Ukrainian people have been through, another failure would be unforgiveable.

*Conflicts of interest*: None declared.
